# Retraction Note: Phosphor-IWS1-dependent *U2AF2* splicing regulates trafficking of CAR-E-positive intronless gene mRNAs and sensitivity to viral infection

**DOI:** 10.1038/s42003-021-02941-1

**Published:** 2021-12-15

**Authors:** Georgios I. Laliotis, Adam D. Kenney, Evangelia Chavdoula, Arturo Orlacchio, Abdul Kaba, Alessandro La Ferlita, Vollter Anastas, Christos Tsatsanis, Joal D. Beane, Lalit Sehgal, Vincenzo Coppola, Jacob S. Yount, Philip N. Tsichlis

**Affiliations:** 1 Department of Cancer Biology and Genetics, The Ohio State University College of Medicine Columbus, Columbus, OH 43210 USA; 2 The Ohio State University Comprehensive Cancer Center, Columbus, OH 43210 USA; 3 University of Crete, School of Medicine, Heraklion Crete, 71500 Greece; 4 Department of Microbial Infection and Immunity and Infectious Diseases Institute, The Ohio State University, Columbus, OH 43210 USA; 5 Department of Clinical and Experimental Medicine, Bioinformatics Unit, University of Catania, Catania, 95131 Italy; 6 Tufts Graduate School of Biomedical Sciences, Program in Genetics, Boston, MA 02111 USA; 7 Institute of Molecular Biology and Biotechnology, Heraklion, Crete, 70013 Greece; 8 Department of Surgery, Division of Surgical Oncology, Columbus, OH 43210 USA; 9 Department of Medicine, Division of Hematology, The Ohio State University, Columbus, OH 43210 USA; 10 Present Address: Sidney Kimmel Comprehensive Cancer Center and Department of Oncology, Johns Hopkins School of Medicine, Baltimore, MD 21205 USA

**Keywords:** Non-small-cell lung cancer, RNA transport

Retraction to: *Communications Biology* 10.1038/s42003-021-02668-z, published online 11 October 2021.

The authors are retracting this Article as irregularities were found in the data that indicate the splicing of the U2AF2 exon 2 does not occur as reported in the Article. The irregularities call into question the conclusions and undermine our full confidence in the integrity of the study. The authors therefore wish to retract the Article.

All of the authors agree with the retraction.

